# The interplay of autonomous learning behavior, academic buoyancy and second language demotivation in the GenAI-assisted informal digital learning of English

**DOI:** 10.3389/fpsyg.2026.1810246

**Published:** 2026-05-21

**Authors:** Ni Cheng, Jing Zhang

**Affiliations:** 1 School of Mathematics and Information Technology, Yuncheng University, Yuncheng, China; 2 School of Educational Studies, Universiti Sains Malaysia, Penang, Malaysia

**Keywords:** academic buoyancy, autonomous learning, demotivation, generative AI, informal digital learning of English

## Abstract

**Introduction:**

Numerous studies identified the positive effects of GenAI tools in the formal language learning settings, but further efforts are still recommended to explore the use of GenAI tools in the Informal Digital Learning of English (GenAI-IDLE). To address the research gap, this study intends to figure out how GenAI-IDLE alleviate the second language (L2) demotivation through autonomous learning behavior and academic buoyancy based on the Basic Psychological Needs Theory and the Organismic Integration Theory.

**Methods:**

The approach of structural equation modelling was adopted to analyze the questionnaire data among first-year college students. Confirmatory factor analysis and serial mediation analysis were conducted through Mplus 8.3.

**Results:**

The findings revealed positive effects of GenAI-IDLE on autonomous learning behavior and academic buoyancy, along with a negative correlation between GenAI-IDLE and L2 demotivation. Besides, the chain mediation of autonomous learning behavior and academic buoyancy was identified to mitigate the L2 demotivation through GenAI-IDLE activities.

**Discussion:**

Informed by these results, the present study offers both theoretical and practical implications. It not only draws on the Basic Psychological Needs Theory and the Organismic Integration Theory to explain the role of GenAI-IDLE practice, but also sheds light on optimising the design of GenAI-IDLE activities to address the demotivation issue from both behavioral and psychological perspectives.

## 1 Introduction

The acquisition of linguistic skills cannot merely rely on the classroom instruction from teachers, but also requires enormous time and efforts of second language (L2) learners through out-of-class learning activities with the support of modern technologies, or the informal digital learning of English (IDLE) activities (Lai and Gu, 2011). In recent years, research on IDLE has attracted wide attention from international scholars due to its complement role of the formal classroom instruction (Soyoof et al., 2023; Kusyk et al., 2025; Guo and Lee, 2023). In the IDLE context, L2 learners could acquire receptive skills such as memorizing vocabularies through mobile Apps, and also enhance productive skills, such as posting English blogs on social media platforms (Lee and Drajati, 2019). The thriving development of GenAI technology further empowers L2 learners with promising opportunities to carry out IDLE activities (Liu et al., 2025). GenAI tools, such as ChatGPT, can be employed to practice speaking or writing skills based on the immediate and personalized feedback (Teng, 2025; Zhang et al., 2026b). Nevertheless, the effects of GenAI-assisted IDLE activities are not limited in the improvement of linguistic competence, but can be also extended to the behavioral, psychological and motivational aspects (Wang et al., 2026; Gao et al., 2025).

Recent studies offer solid evidence that the process of interacting with GenAI tools contributes to psychological factors, such as autonomous learning, academic buoyancy and (de)motivation (Shi and Shakibaei, 2025). In a recent systematic review, Han et al. (2025) identified autonomous learning and motivation as the most obvious benefits of using GenAI tools based on 68 literature papers. Khasawneh et al. (2024) also recognized the positive role of AI-assisted language assessment in boosting the academic buoyancy of language learners after the intervention of using ChatGPT in their experiment. Based on the structural equation modeling and psychological network analysis, Wang et al. (2026) illustrated the motivation, particularly the ideal L2 self, was closely connected with positive emotions and engagement in the GenAI-IDLE activities. However, the motivation of L2 learning is not static, and the demotivation issue would become a major challenge in the AI-assisted language learning process (Namaziandost et al., 2023). Therefore, it is essential to explore the mechanism of taking part in GenAI-IDLE activities to mitigate academic demotivation, along with the serial mediation of autonomous learning behavior and academic buoyancy.

There is also insufficient focus on the IDLE practice supported by GenAI technologies among learners with low English scores (Zhang and Liu, 2025). For Chinese college learners, those with scores below 90 points (out of 150) in the National Matriculation English Test (NMET) are classified into learners with low English scores (Zhang and Bournot-Trites, 2021; Dong, 2022). According to Shi (2025), there exists a higher degree of consistency between English achievement test results and their NMET scores among first-year college students. In addition to weak English proficiency, these students also develop a sense of ineptitude and motivational vulnerability for lack of interest or disappointing learning experiences in the middle schools, thereby increasing their susceptibility to L2 demotivation (Li, 2021; Tang and Hu, 2022). They are also confronted with struggles, such as decreasing level of emotion regulation in face of repeated academic setbacks, or less intention of autonomous learning behaviors in subsequent English learning at colleges (Namaziandost et al., 2023; Zhang and Osman, 2024). Since prior studies elaborated the benefits of GenAI tools in promoting autonomous learning, academic buoyancy and motivation, it is warranted to examine whether GenAI-IDLE activities function as a starting point to reducing the process of L2 demotivation, with the serial mediation path of autonomous learning behavior and academic buoyancy.

Against this background, the present study draws on two mini-theories of the Self-Determination Theory (SDT) as an overarching explanatory framework, including the Basic Psychological Needs Theory and the Organismic Integration Theory. Specifically, the personalized context and immediate feedback from the GenAI-IDLE activities could empower L2 learners with sufficient autonomy, competence and relatedness, which aligns with the tenet of the Basic Psychological Needs Theory (Gao et al., 2025; Zadorozhnyy and Lee, 2025a). The frequent engagement in GenAI-IDLE activities also demonstrates the autonomous learning behaviors of L2 learners, and strengthens the capacity to cope with routine academic difficulties (He, 2025). On a different note, the serial mediation mechanism from GenAI-IDLE to demotivation through autonomous learning behavior and academic buoyancy could be explained with the Organismic Integration Theory. The motivation dynamics also exist in the AI-assisted language learning environment, particularly when learners foster autonomous learning behavior and buoyancy (Teng, 2025). If learners’ needs are adequately met through GenAI-IDLE, they would prefer autonomous learning behavior and become buoyant with daily academic challenges, which drives learners from the motivation continuum toward internal regulation. In this case, learners with low English scores could foster a high level of academic buoyancy to bounce back from regular learning setbacks and ultimately buffer against the emergence of demotivation (Noels et al., 2019; Shi and Shakibaei, 2025).

Therefore, the present study examines whether GenAI-IDLE activities are associated with L2 demotivation among Chinese students with low English scores. Moreover, it tests a serial mediation model in which autonomous learning behavior and academic buoyancy jointly mediate the relationship between GenAI-IDLE and L2 demotivation. Through exploring a vulnerable learner group, a negative motivational outcome, and a theoretically informed mediation pathway, the research seeks to provide a more differentiated understanding of the connections between GenAI-IDLE and L2 demotivation.

## 2 Literature review

### 2.1 GenAI-IDLE and L2 demotivation

The motivation of L2 learning is difficult to sustain, especially for the students with academic challenges who are more likely to encounter with the demotivation issue (Falout et al., 2009; Ren and Abhakorn, 2022). Demotivation can be conceptualized as “a negative process that diminishes or reduces an individual’s motivation associated with an ongoing action or a behavioral intention” (Dörnyei and Ushioda, 2021). When it comes to the language learning, the demotivation can be attributed to internal or external reasons, such as lacking interests, experiences of failures, learning materials, classroom environment, characteristics of classes, and teacher behaviors (Kikuchi, 2015). Particularly, the teacher-centered learning constituted the primary factor of L2 demotivation in the Asian context (Soviana, 2018; Li, 2021). The persisting issue of demotivation is detrimental to the learners’ engagement in the L2 learning, impairs their enthusiasm about enhancing linguistic skills, and hinders the long-term academic success (Namaziandost et al., 2023). In this case, it is essential to cope with the demotivation issue with effective strategies to help students get through such challenge of language acquisition.

The incorporation of GenAI tools into L2 learning is beneficial for strengthening motivation. According to the systematic review of 68 literature papers, Han et al. (2025) reported that the significant effect of utilizing GenAI technology lies in the improvement of learning motivation. Empirical studies also revealed the significant relationship between the use of GenAI tools and (de)motivation in the context of both formal and informal language learning (Liu et al., 2024; Jiang et al., 2025; Shi and Shakibaei, 2025). By contrast, other studies found no significant impact of GenAI-assisted learning on motivation, which potentially leads to the metacognitive laziness (Fan et al., 2025). The longitudinal study from Teng (2025) demonstrated the curvilinear trajectories of motivation changes in the form of rapid initial growth and then gradual plateaus based on the machine learning analysis. Even with numerous relevant studies, it is still unclear about whether GenAI-IDLE activities could mitigate the L2 demotivation. In this case, this research develops the first hypothesis as follows:

*Hypothesis 1 (H1)*: GenAI-IDLE activities negatively predict L2 demotivation.

### 2.2 Autonomous learning behavior and academic buoyancy in GenAI-IDLE

Autonomous learning refers to “an internal capacity of the learners to take charge of, responsibility for, or control over one’s own learning,” and highlighted the autonomous control over abilities, psychology and behaviors (Benson, 2011; Chong and Reinders, 2025). According to Benson (2011), autonomous learning behavior highlights the effective learning management of goals, materials, and learning pace, which can be more easily observed to validate the learner autonomy than psychological and capacity dimensions (Benson, 2011). Although it is highly related to self-regulated learning, the self-regulated learning focuses on the cognitive, metacognitive, and behavioral processes through which learners plan, monitor, evaluate, and adjust their learning strategies in response to different task demands (Zimmerman, 2000). Therefore, this study adopts the autonomous learning behavior as one of critical variables to investigate the effects of GenAI-IDLE. For students with low English scores, such as vocational college learners, they are reluctant to autonomously seek opportunities and resources of authentic L2 experience outside the classroom for lack of learning motivation (Zhang and Liu, 2025; Zhang and Osman, 2024). With a view to this dilemma, the proliferating use of GenAI tools has the potential to promote their autonomous learning behavior with immediate feedback to help them address academic challenges in a low-pressure and personalized learning environments. The evidence in recent literature papers illustrates that the use of GenAI tools in the formal language learning could promote L2 learners to implement self-paced learning, monitor learning process, assess their learning, which suggests the autonomous learning behavior changes for better (Wu and Wang, 2025). Similarly, Sayed et al. (2024) reported a significant increase of learner autonomy after the intervention of utilizing AI-assisted speaking tools, and participants obtained autonomous and efficient language learning experiences due to better control over their learning results. The behavior of autonomous learning also closely correlates with the IDLE activities when L2 learners take initiative in acquiring language skills with the support of technological tools (Lai and Gu, 2011; Benson, 2011; Zadorozhnyy and Lee, 2025b). Thus, it can be hypothesized that GenAI-IDLE activities have a positive effect on autonomous learning behavior.

On a different note, academic buoyancy represents the learner’s capacity to overcome learning setbacks or challenges during the process of language learning (Yun et al., 2018; Wang and Hui, 2024). Buoyant learners could bounce back and step forward in face of challenges, such as exams or deadline of homework assignments (Martin and Marsh, 2008; Hedeshi and Ghanizadeh, 2025), and ultimately promotes the English learning achievement (Zhang et al., 2026a). For students with low English scores, the explorations about improving their academic buoyancy is of great importance, because the high level of academic buoyancy helps them sustain efforts to the L2 learning process and take measures in adjusting learning strategies, instead of being trapped with frustrations or disappointment due to failure of exams or unsatisfactory learning environments (Granziera et al., 2024). Recent studies revealed the positive effect of AI-assisted language learning on improving the academic buoyancy (Sayed et al., 2024; Khasawneh et al., 2024; Namaziandost, 2025). For instance, Khasawneh et al. (2024) indicated that AI-assisted language assessment with the use of ChatGPT could boost the academic buoyancy of language learners from private language institutions in Saudi Arab based on a 10-month experiment. Sayed et al. (2024) also found a noticeable increase of academic buoyancy among students studying at an Ethiopian university after the experiment of incorporating ChatGPT into enhancing assessment and feedback mechanisms for speaking skills. However, there are limited studies on academic buoyancy and the use of GenAI tools in the IDLE context, particularly among low-achieving learners (Sayed et al., 2024). Therefore, the second hypothesis is proposed.

*Hypothesis 2 (H2)*: GenAI-IDLE activities positively predict autonomous learning behavior and academic buoyancy.

### 2.3 The mediating role of autonomous learning behavior

Autonomous learning behavior is closely related to active management of the learning process, including setting goals, monitoring progress, reflecting on performance, and adjusting learning strategies in response to task demands (Benson, 2011; Hsieh and Hsieh, 2019). In IDLE settings, this capacity is especially important because learners are expected to select resources and regulate their engagement beyond teacher-directed instruction. Previous research has shown that learners who engage more actively and strategically in IDLE are better able to choose materials aligned with their proficiency levels and learning needs (Zadorozhnyy and Lee, 2025a). Wang and Littlewood (2021) highlighted such freedom or autonomy of choosing preferred or interesting learning materials was crucial to remotivate L2 learners. This is also the case in the IDLE activities supported by GenAI technologies (Wu and Wang, 2025; Gao et al., 2025). The affordance from GenAI-IDLE activities can also supplement the shortcomings in textbook content, teacher instruction and classroom learning environment that potentially demotivated the second language acquisition in the prior studies (Li, 2021; Ren and Abhakorn, 2022). Recent studies also suggested that AI-assisted language learning enhanced learners’ sense of control, facilitate more individualized learning management, and support more self-initiated engagement with language tasks (Sayed et al., 2024). In this sense, GenAI-IDLE may be associated with lower L2 demotivation because it encourages learners to engage in more autonomous and strategically managed learning behavior. Accordingly, the following hypothesis is proposed:

*Hypothesis 3 (H3)*: Autonomous learning behavior mediates the relationship between GenAI-IDLE activities and L2 demotivation.

### 2.4 The mediating role of academic buoyancy

In addition to autonomous learning behavior, the present study regards academic buoyancy as another variable to mediate the effects of GenAI-IDLE on mitigating L2 demotivation. In language learning, such setbacks may include unsatisfactory test scores, discouraging learning environment, or ongoing difficulties, which constitute major demotivating factors among students with low English scores (Albalawi and Al-Hoorie, 2021; Ren and Abhakorn, 2022). To overcome these setbacks, the external support from GenAI tools, could be a potential solution to help L2 learners become buoyant and less demotivated (Namaziandost, 2025; Shi and Shakibaei, 2025). By providing flexible and personalized interaction, GenAI-IDLE could help L2 learners gain the confidence of overcoming the regular learning difficulties, and hereby remotivate their effort based on immediate feedback in the out-of-class learning setting (Datu and Yang, 2021; Xu and Wang, 2024). This process is critical for students with low English scores who are often more susceptible to frustration in conventional learning settings (Qian and Qiu, 2026). Yet, rare explorations have been made to investigate the role of academic buoyancy in mediating the relationship between GenAI-IDLE and L2 demotivation. Based on this reasoning, this study proposes the following hypothesis:

*Hypothesis 4 (H4)*: Academic buoyancy mediates the association between GenAI-IDLE activities and L2 demotivation.

### 2.5 The chain mediation of autonomous learning behavior and academic buoyancy

The Self-Determination Theory (SDT) serves as the overarching theoretical lens of the present study, particularly two mini-theories, including Basic Psychological Needs Theory (BPNT) and Organismic Integration Theory (OIT). From the perspective of BPNT, the GenAI-IDLE activities could function as a starting point to support autonomy, competence and relatedness (Gao et al., 2025; Wu and Wang, 2025). Specifically, learners could take control over their learning materials, strategies and progress, and enhance linguistic competence through both receptive and productive skills in the context of informal language learning (Zadorozhnyy and Lee, 2025a). Moreover, the relatedness can change for better when L2 learners build connections with peers with similar interests in online communities or have frequent interactions with GenAI tools based on immediate feedback (Gao et al., 2025). Among these affordances, autonomy support from GenAI-IDLE activities is particularly relevant to the present study when learners with low English scores carry out autonomous learning behavior and develop academic buoyancy to overcome learning difficulties.

On the other hand, OIT focuses on the continuum from lack of motivation to forms of extrinsic motivation and intrinsic motivation, and illustrates the quality of motivation that stems from external incentives to improve learning outcomes in different learning settings (Ryan and Deci, 2017; Al-Hoorie et al., 2025). Within this continuum, affordances from with GenAI tools, could help learners transform from the external regulation (autonomous learning behavior) into internal regulation, thereby benefiting their psychological well-being and boosting the motivation (Pelletier and Rocchi, 2023; Schweder et al., 2026). In the present study, this tendency is reflected behaviorally in autonomous learning behavior, which helps learners plan, monitor, and adjust their learning more effectively in GenAI-IDLE activities (Sayed et al., 2024). Over time, such external regulation may strengthen academic buoyancy, that is, learners’ capacity to cope with routine academic setbacks without becoming overwhelmed or disengaged (Kim et al., 2017; Gao et al., 2025). Greater academic buoyancy from the interaction with GenAI tools could in turn reduce learners’ susceptibility to the demotivation in the language learning process (Datu and Yang, 2021; Xu and Wang, 2024; Namaziandost, 2025). In this sense, the OIT provides a coherent rationale for the effects of GenAI-IDLE on mitigating L2 demotivation through the serial mediation of autonomous learning behavior and academic buoyancy.

**Figure 1:**
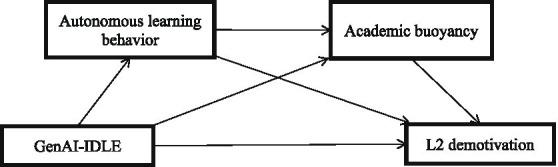
Hypothesized serial mediation model of the variables.

Consistent with the SDT-informed framework, GenAI-IDLE could support more autonomous learning behavior, and foster learners’ capacity to cope with routine academic difficulties. GenAI-IDLE activities offer a more autonomy-supportive learning context based on BPNT, while such support could alleviate the L2 demotivation by helping L2 learners more autonomous and buoyant, as illustrated by the OIT. In this sense, autonomous learning behavior can be viewed as a more proximal form of learning regulation, whereas academic buoyancy reflects a more adaptive response pattern that may develop from such regulation over time. Accordingly, GenAI-IDLE could lower L2 demotivation through a sequential pathway in which learners first engage in autonomous learning behavior and then become better buoyant to handle everyday setbacks (Figure 1). Therefore, the following serial mediation hypothesis is proposed:

*Hypothesis 5 (H5)*: Autonomous learning behavior and academic buoyancy serially mediate the effect of GenAI-IDLE activities on L2 demotivation.

## 3 Methodology

### 3.1 Participants

This study recruited first-year Chinese students at one vocational college in their first semester as participants through the convenience sampling method and then further screened based on the inclusion and exclusion criteria. Since scores of National Matriculation English Test remained a proximal indicator of prior English attainment for first-year Chinese college students, the participants were screened based on their scores below 90 points. In addition, only students with prior GenAI-IDLE experience were included, as participants without such experience would not have been able to provide meaningful responses to the variables of the study. Students who did not meet these criteria were excluded from the final sample. The sample size was determined based on calculations with the G*Power 3.1. The minimum sample size is 128 when a structural equation modeling is conducted with medium effect size (*f*^2^ = 0.15), significance level α = 0.05, and statistical power (1-β) = 0.8 (Faul et al., 2009). Finally, the sample consisted of 338 students, including 151 males and 187 females, and their average age reached 18.4 years old. Participants majored in disciplines such as Mechanical Engineering, Software Engineering, Internet of Things Engineering, Animal Science, and Modern Agriculture. All participants were enrolled in the compulsory course of “College English” as part of their first-year curriculum.

### 3.2 Instruments

To measure the constructs, this study employed four established instruments that were adapted to the GenAI-IDLE and Chinese EFL context. As the present study adopts an SDT-informed framework, these four scales were selected due to the close relevance with behavioral and psychological variables examined in this study. Except for the GenAI-IDLE scale, the other three scales was originally in English, and two experts in language translation were invited to review the Chinese translation of three scales to ensure the content validity.

Firstly, the bilingual version of GenAI-IDLE scale, adopted from Liu and Ma (2024), was employed to evaluate participants’ frequency and diversity of using GenAI tools in informal digital English learning contexts in five-point Likert format. To capture specific learners’ activities and engagement levels in AI-assisted environments, the scale took reference from studies on IDLE (Lee and Drajati, 2019; Lee and Lee, 2021). Moreover, this eight-item questionnaire is reliable with the value of Cronbach’s α at 0.956. The scale also showed acceptable validity in a one-factor CFA (χ^2^/df = 3.90, RMSEA = 0.09, SRMR = 0.02, CFI = 0.98, TLI = 0.97).

The autonomous learning behavior scale with six items is originated from the version used in Sanprasert (2010) and Tsai (2021), and reflects the autonomous measures that learners take in regular learning activities. The value of Cronbach’s α is 0.843, indicating the good reliability of this five-point Likert scale and acceptable construct validity (χ^2^/df = 3.06, RMSEA = 0.08, SRMR = 0.03, CFI = 0.98, TLI = 0.96).

The four-item academic buoyancy scale, formulated by Martin and Marsh (2008), could reflect the extent to which students can manage to deal with setbacks and challenges in their regular learning. The five-point Likert scale has good reliability in this study (Cronbach’s α = 0.835), and its one-factor CFA indicated acceptable construct validity (χ^2^/df = 7.95, RMSEA = 0.14, SRMR = 0.03, CFI = 0.97, TLI = 0.92).

The L2 Demotivation Scale, including five items developed by Albalawi and Al-Hoorie (2021), is employed to measure the degree of demotivation in L2 learning. This scale is presented in the five-point Likert format with good reliability in this research (Cronbach’s α = 0.906), and acceptable validity (χ^2^/df = 7.09, RMSEA = 0.13, SRMR = 0.03, CFI = 0.97, TLI = 0.95).

### 3.3 Procedure

The research procedures adhered strictly to the guidelines of the Declaration of Helsinki, and researchers obtained the ethical approval from the institutions before the data collection. Data were collected between November 2024 and December 2024. To ensure clarity and accessibility, all survey instruments were administered in the participants’ native language (Chinese). Before answering questionnaires, students were informed of the research objectives, and got aware that their data would be only used for research purposes with confidentiality. Besides, all participants voluntarily filled out the questionnaires, and understood that they could withdraw the research at any time.

### 3.4 Data analysis

Before performing the structural equation modeling, data cleaning, normal distribution analysis and descriptive analysis were conducted with SPSS 25. Specifically, 15 cases were deleted due to outliers with z-scores beyond −3.3 and +3.3, and the expectation–maximization algorithm was imputed to address the issue of missing data. Normal distribution was examined with the skewness (−2 to +2) and kurtosis (−7 to +7). In addition, researchers also computed other descriptive data, such as mean value, standard deviation and correlation.

The measurement models, along with the variables relationships, were analyzed with the use of Mplus 8.3. The study evaluated the model fit based on several indicators, such as confirmatory fit index (CFI), Tucker-Lewis index (TLI), Standardized Root Mean Square Residual (SRMR), and root mean square error of approximation (RMSEA). As presented in Table 1, the results of several indicators showed an excellent model fit (χ^2^/df = 2.144, CFI = 0.954, TLI = 0.948, RMSEA = 0.058, and SRMR = 0.045) (Kline, 2011). Besides, indirect relationships were assessed with the bootstrapping procedures with 5,000 samples.

**Table 1:** Goodness of fit.

Criteria		Threshold		
		Terrible	Acceptable	Excellent
CMIN	480.205			
Df	224			
CMIN/df	2.144	>5	>3	>1
RMSEA	0.058	>0.08	<0.08	<0.06
CFI	0.954	<0.9	>0.9	>0.95
TLI	0.948	<0.9	>0.9	>0.95
SRMR	0.045	>0.1	>0.08	<0.08

## 4 Results

### 4.1 Descriptive statistics and correlations

Table 2 outlines the descriptive statistics (e.g., mean values, standard deviations, skewness, kurtosis), and correlation analysis of all variables involved in this study. As illustrated, positive associations are identified between GenAI-IDLE and autonomous learning behavior (*r* = 0.560, *p* < 0.001), and also buoyancy (*r* = 0.521, *p* < 0.001). On a different note, GenAI-IDLE negatively predicts the demotivation (*r* = −0.589, *p* < 0.001). Moreover, it can be seen that demotivation has negative relationships with autonomous learning behavior (*r* = −0.501, *p* < 0.001), and buoyancy (*r* = −0.567, *p* < 0.001).

**Table 2:** Descriptive statistics and correlations analysis for the variables.

Variables	*M*	SD	1	2	3	4
1. GenAI-IDLE	3.056	0.939	1			
2. ALB	3.639	0.626	0.560^***^	1		
3. Buoyancy	3.167	0.851	0.521^***^	0.462^***^	1	
4. Demotivation	2.689	1.019	−0.589^***^	−0.501^***^	−0.0567^***^	1
Skewness (<±2)	–	–	−0.307	−0.172	0.089	−0.104
Kurtosis (<±7)	–	–	−0.009	1.718	0.352	−0.758

*N* = 338. M, mean; SD, standard deviations; ALB, autonomous learning behavior. ***p < 0.001 (two-tailed).

### 4.2 Test of direct effects

To assess the serial mediation model, structural equation modeling was performed with Mplus 8.3. According to the results in Table 3, GenAI-IDLE significantly predicted autonomous learning behavior (β = 0.628, *z* = 12.704, *p* < 0.001), and also had a significant positive effect on academic buoyancy (β = 0.384, *z* = 4.874, *p* < 0.001). The positive correlation was also identified between autonomous learning behavior and academic buoyancy (β = 0.316, *z* = 3.598, *p* < 0.001). However, both autonomous learning behavior (β = −0.171, *z* = −2.342, *p* < 0.05) and academic buoyancy (β = −0.394, *z* = −5.407, *p* < 0.001) were negatively associated with L2 demotivation. GenAI-IDLE also negatively predicted L2 demotivation (β = −0.286, *z* = −3.924, *p* < 0.001). Besides, it is noted that the model accounted for 39.4% of the variance in autonomous learning behavior, 39.9% in academic buoyancy, and 53.4% in L2 demotivation.

**Table 3:** Path estimates and explained variance for the serial mediation model.

Antecedents	Outcomes	*R* ^2^	β	S. E	*z*	*p*-value
ALB	GenAI-IDLE	0.394	0.628	0.049	12.704	0.000
Buoyancy	GenAI-IDLE	0.399	0.384	0.079	4.874	0.000
	ALB		0.316	0.088	3.598	0.000
Demotivation	ALB	0.534	−0.171	0.073	−2.342	0.019
	Buoyancy		−0.394	0.073	−5.407	0.000
	GenAI-IDLE		−0.286	0.073	−3.924	0.000

*N* = 338. ALB, autonomous learning behavior.

### 4.3 Test of chain mediation effects

The computation of mediation effects followed the bootstrapping procedures with 5,000 samples as well as bias-corrected confidence intervals. According to Table 4, the direct effect of GenAI-IDLE on demotivation was significant (β = −0.286, SE = 0.073, *z* = −3.924, *p* < 0.001, 95% CI [−0.419, −0.131]), so that GenAI-IDLE independently mitigates the negative role of L2 demotivation. Besides, statistical significance was identified when it came to the total indirect effect of GenAI-IDLE on demotivation (β = −0.325, SE = 0.06, *z* = −5.428, *p* < 0.05, 95% CI [−0.458, −0.22]). In terms of the specific indirect pathways, the mediation effect of autonomous learning behavior was significant (β = −0.104, SE = 0.046, *z* = −5.428, *p* < 0.05, 95% CI [−0.194, −0.007]), and it was also the case about the mediation effect through academic buoyancy (β = −0.146, SE = 0.043, *z* = −3.393, *p* < 0.01, 95% CI [−0.246, −0.078]). Thus, the Hypothesis 3 and Hypothesis 4 were validated. Moreover, statistical significance was found with regard to the sequential pathway through both autonomous learning behavior and academic buoyancy (β = −0.075, SE = 0.03, *z* = −2.508 *p* < 0.05, 95% CI [−0.151, −0.031]), which supported the Hypothesis 5. In practical terms, this serial pathway was modest in magnitude, accounting for 12.5% of the total effect. These results confirm that both autonomous learning behavior and academic buoyancy serve as sequential mediators in the relationship between GenAI-IDLE and demotivation, while also affirming the direct effect of GenAI-IDLE on L2 demotivation.

**Table 4:** Total, direct, and indirect effects of GenAI-IDLE on demotivation.

Path	β	S. E	*z*	*p*-value	95% CI
Total effect	−0.601	0.055	−10.95	0.000	[−0.739, −0.467]
Direct effect (GenAI-IDLE → Demotivation)	−0.286	0.073	−3.924	0.000	[−0.478, −0.083]
Total indirect effects	−0.325	0.06	−5.428	0.000	[−0.503, −0.183]
GenAI-IDLE → ALB → Demotivation	−0.104	0.046	−2.276	0.023	[−0.220, −0.013]
GenAI-IDLE → Buoyancy → Demotivation	−0.146	0.043	−3.393	0.001	[−0.284, −0.055]
GenAI-IDLE → ALB → Buoyancy → Demotivation	−0.075	0.03	−2.508	0.012	[−0.179, −0.022]

*N* = 338. ALB, autonomous learning behavior.

## 5 Discussion

This study aimed to unveil the serial mediation model between the GenAI-IDLE, autonomous learning behavior, academic buoyancy and L2 demotivation. The results of first hypothesis demonstrated a negative correlation between GenAI-IDLE and L2 demotivation. To put it differently, the use of GenAI tools in the IDLE context could buffer the negative effect of demotivation. By providing immediate and individualized feedback, GenAI tools can simulate authentic human-like communication, thereby stimulating their learning interest and boosting confidence (Liu et al., 2025). Moreover, the affordance from GenAI-IDLE activities can also supplement the shortcomings in textbook content, teacher instruction and classroom learning environment that potentially demotivated the second language acquisition in the prior studies (Li, 2021; Ren and Abhakorn, 2022; Shi and Shakibaei, 2025). The supportive learning environment from the GenAI-IDLE activities would help students with low English scores remotivate their efforts in L2 learning (Wang and Littlewood, 2021). Similar findings were also reported in a recent study from who illustrated the negative associations between AI-assisted language learning and academic demotivation (Shi and Shakibaei, 2025). The findings also offered a reverse perspective to complement the current studies on enhancing motivation with the GenAI tools (Deng et al., 2025; Rezai and Goodarzi, 2025; Zheng and Xiao, 2024).

In addition to the direct effects, this research also identified three indirect pathways that mediated the relationship between GenAI-IDLE and L2 demotivation. Above all, the mediation role of autonomous learning behavior supported the third hypothesis, which suggested that autonomous learning behavior from the GenAI-IDLE activities in turn alleviate the negative influence of L2 demotivation. The findings also corroborate the prior studies to illustrate the role of autonomous behavior when L2 learners interacted with GenAI tools in both formal and informal settings (Wu and Wang, 2025). According to the BPNT, the adaptive learning afforded by GenAI tools enabled L2 learners to monitor their progress, identify learning issues and then adjust learning strategies based on instant feedback, which in turn reinforces the motivation in second language acquisition (He, 2025; Sayed et al., 2024; Zadorozhnyy and Lee, 2025a). In this regard, the mitigating effect of GenAI-IDLE activities on demotivation cannot be separated from autonomous learning behavior, such as setting goals, monitoring learning progress, making AI-assisted assessment and adjusting learning strategies.

The fourth hypothesis was also supported when it comes to the mediation effect of academic buoyancy on undermining the negative correlation between GenAI-IDLE and L2 demotivation. Such finding not only aligns with the recent studies on the effect of GenAI tools on academic buoyancy (Sayed et al., 2024; Khasawneh et al., 2024; Namaziandost, 2025), but also underscores the significant role of academic buoyancy in alleviating the demotivation process from a converse perspective (Datu and Yang, 2021; Fathi et al., 2025). The interconnected relationships among these variables can be attributed to the effectiveness of GenAI tools in supporting the problem-solving skills, tenacity, and flexibility through immediate feedback. For instance, Sayed et al. (2024) revealed that the regular use of ChatGPT helped the language learners boost their confidence in speaking proficiency, tackle with academic problems, and bounce back from failures rooted in daily learning activities (Gao et al., 2025). In light of this finding, the incorporation of different GenAI tools is recommended to foster the development of positive psychological factors when researchers put focus on developing linguistic skills of students with low English scores.

The result of the fifth hypothesis demonstrated the chain mediation of autonomous learning behavior and academic buoyancy, suggesting that the significant role of both behavioral and psychological constructs in the GenAI-IDLE activities. Nevertheless, the serial effect size in the hypothesized model was relatively small, suggesting that this pathway should be interpreted as a modest explanatory mechanism to alleviate L2 demotivation through GenAI-IDLE. GenAI-IDLE activities empower L2 learners to autonomously select learning materials in line with their interests and abilities, evaluate their own learning progress, overcome learning difficulties with immediate feedback from GenAI tools (Wu and Wang, 2025). The constant interaction with GenAI tools subsequently promotes academic buoyancy among low-achieving students who fulfill their psychological needs of autonomy, relatedness and competence through the GenAI-IDLE activities (Sayed et al., 2024; Gao et al., 2025). In this case, GenAI-IDLE activities could buffer the negative effects of demotivation due to the enhancement of autonomous learning behavior and academic buoyancy, which offered a nuanced understanding of alleviating the demotivation issue from both behavioral and psychological perspectives based on GenAI-IDLE activities.

## 6 Implications

Informed by these results and discussions, the research has following implications for theoretical and pedagogical considerations. Theoretically, this research provides an SDT-informed account of how GenAI-IDLE activities are associated with mitigating L2 demotivation. The present study draws on BPNT and OIT to interpret how GenAI-IDLE activities offer a supportive learning context and how such support promotes autonomous learning behavior, and subsequently, in academic buoyancy. In this sense, the findings suggest that autonomous learning behavior and academic buoyancy could serially link the connection between GenAI-IDLE and lower susceptibility to L2 demotivation. Furthermore, the study contributes to the literature by linking GenAI-IDLE with both behavioral and adaptive psychological changes in L2 learning. The findings indicate that the role of GenAI-IDLE should not be understood merely as technological participation to promote linguistic proficiency, but function as a potential pathway to help learners manage their learning, cope with routine setbacks, and mitigate demotivation due to prior discouraging experiences.

Practically, this study offers the following pedagogical implications for the language learning and teaching practice. In the first place, incorporating GenAI tools can serve as a catalyst for promoting the autonomous learning behavior through controlling their learning in flexible ways. Learners with low English scores are also recommended to apply GenAI tools into dealing with regular academic difficulties and enhancing problem-solving abilities with the technological support, which strengthens their buoyancy and alleviates the demotivation. On a different note, language teachers are suggested to promote the learner autonomy and academic buoyancy so that students could develop a great sense of control over their L2 learning process both in and outside the classroom. Finally, it is essential for policy makers to formulate guidelines of leveraging GenAI tools, and offer professional training of AI literacy for both teachers and students to help them ethically and critically discriminate the answers generated by GenAI tools. In this vein, L2 learners could benefit from effective strategies of cultivating autonomy and buoyancy to alleviate the demotivation issue, while fostering the independent thinking in the GenAI-IDLE activities.

## 7 Conclusion

Drawing on the BPNT and OIT, this study disclosed the intricate relationships between the GenAI-IDLE, autonomous learning behavior, academic buoyancy and L2 demotivation. The results firstly demonstrated positive effects of GenAI-IDLE on autonomous learning behavior and academic buoyancy, along with a negative correlation between GenAI-IDLE and L2 demotivation. Besides, the chain mediation of autonomous learning behavior and academic buoyancy was identified to mitigate the demotivation through GenAI-IDLE activities. These findings offered SDT-informed investigation of AI-mediated informal learning contexts and suggested that educators and instructional designers can focus on promoting autonomous learning behavior and academic buoyancy among students with low English scores to address their demotivation issues with the assistance of GenAI tools.

Nevertheless, this study has several limitations that should be acknowledged. First, the sample was recruited from one university through convenience sampling, which limits the representativeness of the participants. Moreover, the group of students with low English scores was identified based on Chinese context, namely a self-reported NMET English score below 90 out of 150, which limits the generalibility of the sample. Second, the study did not directly measure basic psychological needs or motivational regulation types, and the GenAI-IDLE scale primarily captured the reported frequency and diversity of learners’ engagement, rather than the quality of engaging in GenAI-ILDE activities, which cannot fully reflect the comprehensive use of GenAI-IDLE in the regular learning activities. Third, the results in this study relied on the cross-sectional and self-reported questionnaires, rather than triangulated data, such as interviews, classroom observations, learning logs, or longitudinal evidence, which restricted a more nuanced understanding of the effects of GenAI-IDLE activities over time.

Future research is recommended to adopt more diverse and triangulated data sources, such as learning logs, interviews, classroom observations, and reflective journals, in order to fully capture learners’ behavioral and psychological experiences in GenAI-IDLE. It would also be valuable to employ other scales of relevant variables, especially validated scales of basic psychological needs and motivational regulation, and to consider contextual variables such as teacher support and learning environment. To improve generalizability, future studies should recruit participants from a wider range of institutions, or L2 learners from diverse cultural and multilingual backgrounds. In addition, longitudinal and intervention-based designs, particularly in blended learning contexts where GenAI-IDLE complements formal instruction, would help provide a more dynamic understanding of how AI-integrated learning contexts influence learners’ motivation, autonomy and buoyancy over time.

## Data Availability

The raw data supporting the conclusions of this article will be made available by the authors, without undue reservation.
